# Supplementary material for: Non-invasive focusing and imaging in scattering media with a fluorescence-based transmission matrix
Source: Nat Commun. 2020 Dec 1;11:6154. doi: 10.1038/s41467-020-19696-8 (PMC7708489; doi:10.1038/s41467-020-19696-8)
Supplement: Supplementary file 2 — Supplementary Information [file 41467_2020_19696_MOESM2_ESM.pdf]

Supplementary Information: Non-invasive focusing and imaging in scattering media with a fluorescence-based transmission matrix

Antoine Boniface,[1] Jonathan Dong,[1,2] and Sylvain Gigan[1]

[1] *Laboratoire Kastler Brossel, Sorbonne Université, École Normale Supérieure-Paris Sciences et Lettres (PSL) Research University, Centre National de la Recherche Scientifique (CNRS) UMR 8552, Collège de France, 24 rue Lhomond, 75005 Paris, France**

[2] *Laboratoire de Physique de l'École Normale Supérieure, Université Paris Sciences et Lettres (PSL), Centre National de la Recherche Scientifique (CNRS), Sorbonne Université, Université Paris-Diderot, Sorbonne Paris Cité, 24 rue Lhomond, 75005 Paris, France.*

Supplementary Note 1: Experimental setup

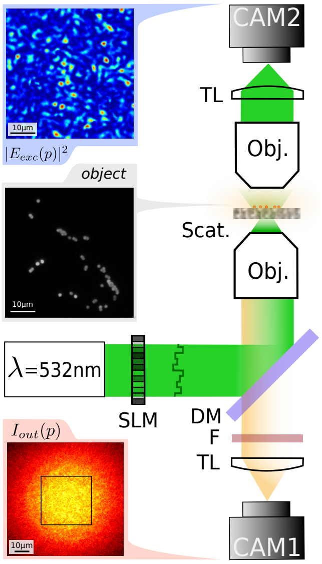

FIG. 1. Scheme of the experimental setup – From top to bottom – $|E_{exc}(p)|^2$ is the illumination speckle in the plane of the fluorescent object recorded on the control camera CAM2. *object* is a fluorescence image of the object obtained on CAM1 without the scattering medium. $I_{out}(p)$ is a typical fluorescent speckle image epi-detected on CAM1. The dark square indicates the typical the cropped region we use as input data for the algorithm. DM: dichroic mirror, TL: tube lens, F: filter, Scat.: scattering medium.

Supplementary Note 2: TM-reconstruction robustness

In this subsection, the experimental data corresponds to the ones presented in the Fig. 2 of the manuscript.

To prove the robustness of our method, we decided to reconstruct T for different ranks $r \in [15; 30]$. We thus run the NMF 16 times which provides 16 pairs of matrices $\{W, H\}$. From $H = |TE_{in}|^2$ and knowing SLM patterns E_{in} we can recover T with phase retrieval. Repeating this procedure for all the 16 matrices H gives rise to 16 different field-TMs of size $r \times N_{SLM}$, that can be used to focus light. As a result, all the TMs whatever their rank r, are able to focus light, Fig. 2b. This proves that the algorithm is robust and does not produce outliers if the rank is not exact. There are only 12 points with no focus at all and almost 94% of the focus spots have

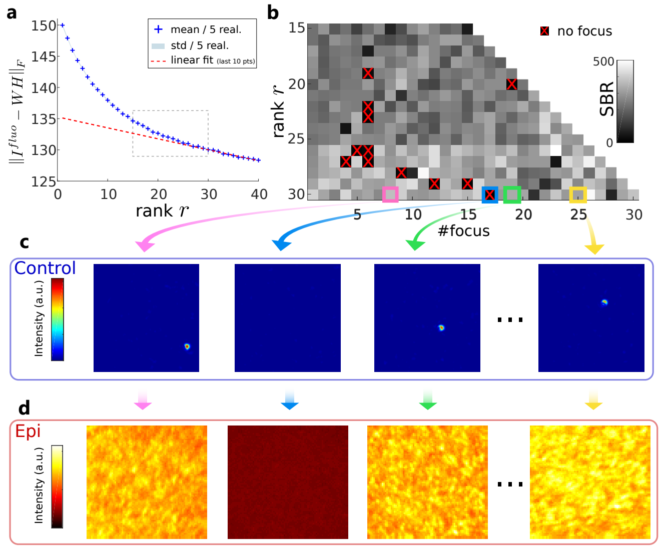

FIG. 2. Experimental Results. (**a**) Frobenius norm of the NMF residual for rank estimation. When the latter is minimized the rank of I_{out} is approximately found. It should correspond to N, the number of targets. Here, we have only a rough estimation of the rank of I_{out}. (**b**) TM reconstruction for different rank $r \in [15; 30]$. Whatever the rank r set for the reconstruction all the 16 TM are able to focus light. The only difference is the number of different spatial positions they can achieve. (**c**) Examples of foci obtained after phase conjugation of T for $r = 30$. Over the 30 lines of T, only one #17, is not well reconstructed since it generates a speckle instead of a focus. (**d**) These spurious data can be tracked, non-invasively, by looking at the corresponding epi-detected fluorescence pattern. Its spatial variance is much lower than the one when the bead is successfully focused.

* antoine.boniface@lkb.ens.fr

an SBR higher than 100. Looking at the variance of the fluorescence patterns on CAM1 enables us to track the points where reconstruction fails using the approach developed in [1]. As done in the manuscript, our strategy consisted of overestimating the rank $r > N$ and removing spurious data afterwards.

Supplementary Note 3: Algorithm Convergence

In the following subsection, an additional simple experiment with only $N = 4$ targets is conducted, in order to study in more details algorithms input requirements and output results.

In the following subsection, we aim at experimentally investigating the different parameters set for the algorithm and how they affect the TM reconstruction. In this particular study, the sample is only made of 4

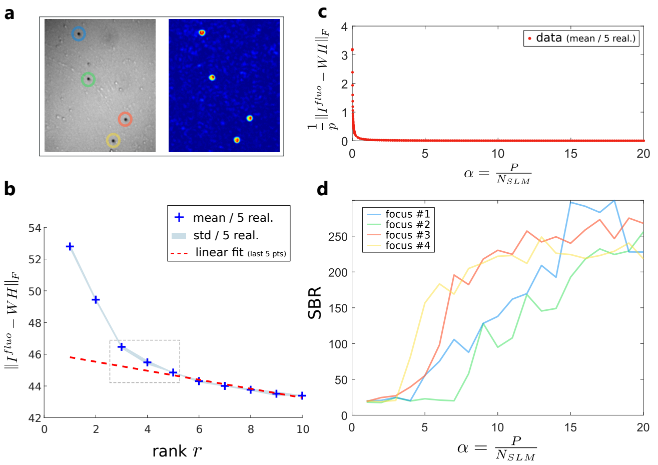

FIG. 3. (**a**) Study of an experimental sample made of 4 targets. Once the TM is reconstructed with our computational pipeline NMF + PR, light is successively focused on all the targets. Only the sum of all the foci is represented. (**b**) Frobenius norm of the NMF residual for rank estimation. (**c**) NMF error with respect to P the number of input data. (**d**) SBR of the generated focus after phase conjugation of T as a function of input data used for the phase retrieval step.

beads which speeds up most of the computational steps. At first, one needs to provide the rank r to factorize I_{out} into W and H. As already mentioned in Methods, this parameter can be estimated from the residual error $\|I_{out} - WH\|_F$ of the NMF itself. A change in the slope is noticed around $r = 4$ which is in good agreement with N. Knowing r, several NMF are done for different number of input patterns p. It shows that only few patterns (< 100) are required to significantly reduce the error: the $r = 4$ eigenvectors of I_{out} are found. The last computational step consists in retrieving the phase from the NMF intensity results to get the transmission matrix. This phase retrieval problem is much more demanding in terms of number of patterns required. To highlight this point, we thus have processed the reconstruction of several TM with a different oversampling ratio $\alpha = P/N_{SLM}$, as this parameter defines the performance of recent phase retrieval algorithms in the large-dimensional limit [2–4]. Then light is focused and TM fidelity is quantified based on the SBR of the generated foci. While in theory the transition is expected for $\alpha \simeq 4-5$, we see with an experimental dataset that SBR > 100 around $\alpha \simeq 10$ and even higher around $\alpha \simeq 15$. We also observe that the convergence is not the same for all the targets, maybe because all the targets are not equally excited due the Gaussian envelope of the illumination.

Supplementary Note 4: TM Refinement via Light Refocusing

Once the computational steps NMF + PR on experimental data I_{out} are performed, a first reconstruction for T is obtained. For several reasons mainly due to experimental noise, T may not be perfectly reconstructed, with two consequences. First the reconstruction may fail: after taking the phase conjugation of those eigenvalues no focus is obtained at all. Second, the reconstruction may generate different eigenvalues focusing on the same target: these are duplicates. In the two cases, one needs to remove the corresponding line to improve the quality of T. Importantly this must be done non-invasively, by only looking at the epi-detected fluorescence. In the first case,

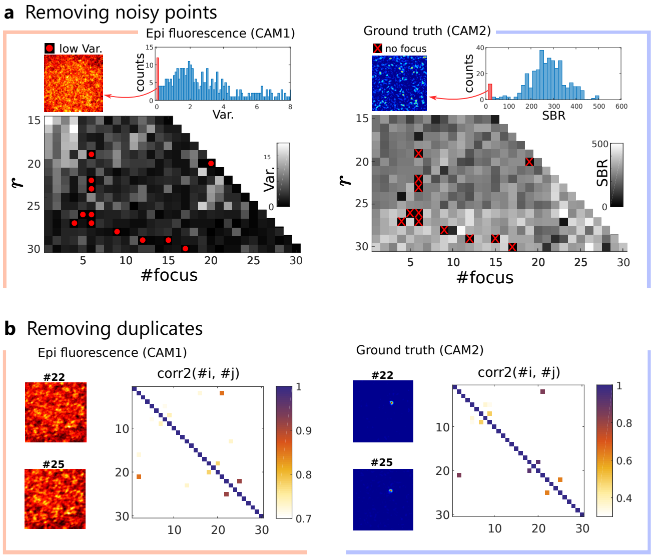

FIG. 4. (**a**) Removing noisy points. When light is not successfully focused in transmission on one of the N targets the corresponding fluorescence speckle pattern has a low spatial variance. (**b**) Removing duplicates. If two lines of the matrix T focus on the same target the emitted fluorescence, is similarly back-scattered by the medium. The epi-detected speckles are thus strongly correlated.

noisy points may be removed by looking at the spatial variance of the fluorescence speckle, as in [1]. In Fig. 4a, we show that the fluorescence speckle having the lowest spatial variance corresponds to the lowest SBR points, i.e. speckle pattern instead of foci. To remove duplicates, we can perform the two-dimensional spatial correlation, Fig. 4b. Here also, there is a good correspondence between what one can see in transmission on CAM2 and what can be epi-detected on CAM1.

Supplementary Note 5: Memory Effect Characterization

The two experiments reported in Fig. 2 and Fig. 3 of the manuscript are conducted with two different scattering media. In the first case, our ability to consistently focus light in transmission is demonstrated through a single diffuser. From the fluorescence fingerprints we can perform some correlation measurements. With CAM2, placed in transmission we know the distance between the sources emitting the former patterns. Altogether, the two information can be used to estimate the memory effect range for the linear fluorescence at 540/560nm. Memory effect for the excitation light at 532nm is expected to be similar. Graph is reported on Fig. 5a. From the graph, we can roughly estimate that memory effect range does not exceed 40µm. The object has a similar size and such memory effect range is enough to recover the full object in one shot using an autocorrelation technique, [5]. In order to show that our non-invasive imaging technique, is not limited to a single memory effect patch, we decided to work in a stronger scattering regime by using two holographic diffusers separated by 0.78mm (the thickness of the diffuser). In this situation, the memory effect range is reduced to ≃20µm, Fig. 5b. We use an extended object, whose typical size is ≃50µm. In this configuration the autocorrelation technique cannot be applied.

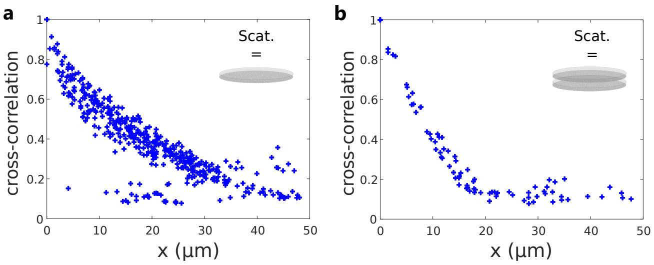

FIG. 5. (**a**)-(**b**) Speckle spatial correlations due to ME inside the scattering medium respectively used for Fig. 2 and Fig. 3.

Supplementary Note 6: PR Necessity for Imaging

To reconstruct the object, as done in Fig. 3 of the manuscript, we exploit the spatial correlations between the fluorescent speckles. In principle, the latter can be retrieved via two different methods: NMF or NMF + PR. In the following, we discuss the differences.

On one hand, the NMF should directly provide the fluorescent eigen-patterns corresponding to each bead, with factor matrix W. However before running the NMF, a Gaussian filter is used to remove structures with spatial frequencies lower than the speckle grain size. The idea is to remove both the detection noise and the fluorescence background envelope. As also discussed in [6], this step seems to be crucial to obtain a reliable demixing through the NMF. As a consequence, the computationally obtained patterns (Fig. 6a) do not contain all the information of fluorescence back-scattering, but only a filtered version. Whereas it is not a problem to retrieve the TMs, it severely impacts the reconstruction of the object.

On the other hand, by reconstructing the matrix T through the NMF + PR pipeline, selective focusing on all the beads can be done thanks to phase conjugation of the TM. While light is focused on the beads, their fluorescence patterns can be non-invasively recorded (Fig. 6b).

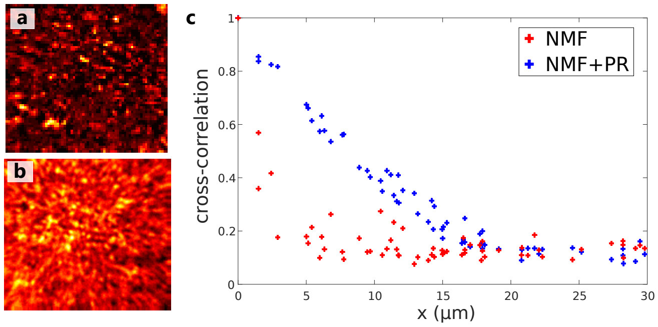

FIG. 6. (**a**) Example of pattern obtained via the NMF. (**b**) Epi-detected fluorescence patterns emitted by the beads when light is focused, NMF + PR. (**c**) Correlation between patterns (obtained via NMF and NMF + PR) as a function of the distance between the fluorescent emitters.

Finally, cross-correlations between patterns obtained through the same method (NMF or NMF + PR) are performed. In the case of NMF, part of the information is missing which explains why patterns have some correlation only if the sources are very close ($<$ 5µm), see Fig. 6c. In the other case, NMF + PR, we directly use the raw patterns measured on the camera, and correlation is maintained over a much larger region ($\sim$ 15µm). Note that the latter is limited by the memory effect of the scattering medium.

Supplementary Note 7: MDS Reconstruction

As described in Fig. 3 of the manuscript, cross-correlation gives access to the translation $\overrightarrow{u_{ij}}$ which corresponds to the relative shifts along the x and y-axis between

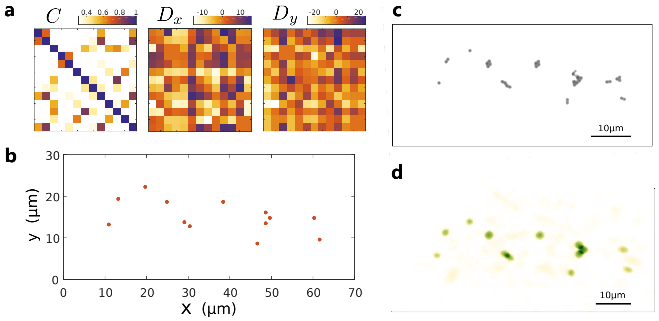

FIG. 7. (**a**) Estimated matrices C, D_x and D_y, from fluorescent speckle cross-correlations. (**b**) MDS reconstruction. (**c**) Ground truth fluorescence image obtained without scattering medium. (**d**) Patch by patch reconstruction.

beads i and j. Once all the pairwise cross-correlations $\{i, j\}$ are calculated, we end up with three matrices:

- C, the correlation matrix which corresponds to the intensity of the off-centered cross correlation peak.
- D_x, the horizontal distance matrix, which stands for the estimated x-axis shift between all the beads.
- D_y, the vertical distance matrix, which stands for the estimated y-axis shift between all the beads.

From there, different strategies for the reconstruction may be considered. The one presented in the manuscript consists in doing the reconstruction patch by patch. One major limitation is that to retrieve the position of one point only the its close neighbour (with strong correlation) are used. Herewith we propose a global approach relying on Multi-Dimensional Scaling (MDS) [7]. In this case, the full distance matrix is used through an optimization algorithm which is more robust to noise than the approach proposed in the manuscript. The stress function is weighted with c_{ij}^{α}, where c_{ij} are the correlation matrix coefficients and reads:

$$stress_{[u_1,u_2,\ldots,u_r]} = \left(\sum_{i \neq j = 1 \ldots r} c_{ij}^{\alpha} (d_{ij} - \|u_i - u_j\|)^2 \right)^{1/2} \tag{1}$$

The optimization is actually run twice, to successively access all the $u_i = x$ and $u_i = y$ coordinates for $i = 1, \ldots, r$, with distance matrix D_x and D_y respectively. Retrieved positions are plotted in Fig. 7a for $\alpha = 6$. This new approach is also in good agreement with the ground truth (**b**). Here, in the case of a relatively simple object, the MDS method provides a reconstruction very similar to the patch by patch approach (**c**). The two images are obtained from the same dataset but through a different reconstruction algorithm.

Supplementary Note 8: Numerical Results with 2-photon Excitation

A major advantage of our approach is that it should be applicable to any incoherent process. In the following, we provide some simulation for 2-photon fluorescence.

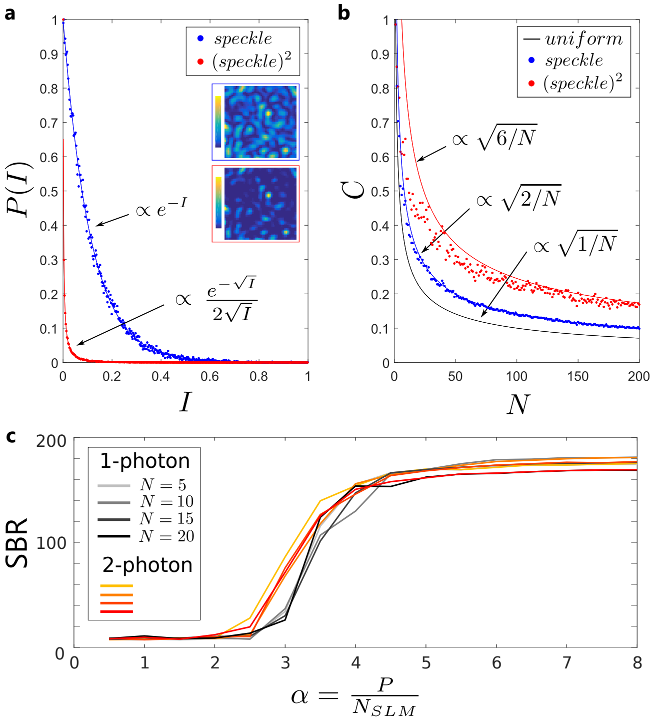

FIG. 8. (**a**) Probability density function respectively derived for, a standard *speckle* defined by intensity I, and a 2-photon speckle, denoted $(speckle)^2$ obtained by squaring the intensity of a standard *speckle*. (**b**) Contrast evolution of fluorescence speckle emitted by N targets. The N targets are excited with different distribution: *uniform*, *speckle* and $(speckle)^2$. (**c**) NMF + PR algorithm is used in the two cases to retrieve matrix T and focus light with a different number of inputs. Whatever the number of targets N the 2-photon configuration proves to be faster in terms of convergence.

This additional non-linearity actually helps our algorithm, since spatial sparsity is even higher. As one can see on Fig. 8a, if speckle intensity is squared, the spatial sparsity is significantly increased. The corresponding speckle is composed by more dark speckle grains and the probability density function is more strongly peaked around zero intensity. The probability to get only few targets excited at once is increased which should make the fluorescence demixing easier. Additionally, such an illumination pattern also improved the contrast of the back-scattered fluorescent. Theory predicts that the contrast scales as $\propto \sqrt{6/N}$ instead of $\propto \sqrt{2/N}$ for 1-photon fluorescence (see after for detailed demonstration), Fig. 8b. It would give the opportunity to increase the number of targets with a similar contrast. These two effects actually help the algorithm to converge. We report on Fig. 8c, simula-

tions for the two different regimes. No noise is added and $N_{SLM} = 256$.

We now derive the contrast of the sum of N non equal strength speckle intensities. We assume that the N individual speckles are statistically independent.

The total intensity of interest is given by the sum $I_s = \sum_{n=1}^{N} I_n$ where I_n has a mean value $\bar{I_n}$. We consider here that each corresponding speckle is fully developed and obeys negative exponential probability distribution. In particular it results in the fact that its mean intensity is equal to its standard deviation, $\sigma_n = \bar{I_n}$. The mean value of the total intensity thus reads $\bar{I_s} = \sum_{n=1}^{N} \bar{I_n}$.

Then, the second moment of the total intensity is $\sigma_s^2 = \sum_{n=1}^{N} \sigma_n^2$ and the contrast of the total intensity is

$$C = \frac{\sigma_s}{\bar{I_s}} = \frac{\sqrt{\sum_{n=1}^{N} \sigma_n^2}}{\sum_{n=1}^{N} \bar{I_n}} \quad (2)$$

If all the N components have an equal mean intensity ($\bar{I_n} = I_0$), equation 2 reduces to the well-known expression $C = \frac{1}{\sqrt{N}}$.

Now we look into the case where the mean intensity $\bar{I_n}$ is not the same for all the N components but rather follows a negative exponential probability distribution. Such speckles are obtained when spatially incoherent beads are excited with a speckle illumination. In this situation the contrast follows $C = \frac{1}{\sqrt{N}} \frac{\sqrt{1/N \sum_{n=1}^{N} \sigma_n^2}}{1/N \sum_{n=1}^{N} \bar{I_n}}$ and tends to $C = \frac{1}{\sqrt{N}} \frac{\sqrt{\int_0^{\infty} I^2 e^{-I} dI}}{\int_0^{\infty} I e^{-I} dI} = \sqrt{\frac{2}{N}}$ when N tends to infinity.

ACKNOWLEDGMENTS

The authors thank Claudio Moretti for fruitful discussions and constructive comments.

ADDITIONAL INFORMATION

This research has been funded by the European Research Council ERC Consolidator Grant (Grant SMARTIES - 724473). S.G. is a member of the Institut Universitaire de France.

[1] A. Boniface, B. Blochet, J. Dong, and S. Gigan, Non-invasive light focusing in scattering media using speckle variance optimization, Optica **6**, 1381 (2019).

[2] C. A. Metzler, M. K. Sharma, S. Nagesh, R. G. Baraniuk, O. Cossairt, and A. Veeraraghavan, Coherent inverse scattering via transmission matrices: Efficient phase retrieval algorithms and a public dataset, in *2017 IEEE International Conference on Computational Photography (ICCP)* (IEEE, 2017) pp. 1–16.

[3] M. Mondelli and A. Montanari, Fundamental limits of weak recovery with applications to phase retrieval, Foundations of Computational Mathematics **19**, 703 (2019).

[4] W. Luo, W. Alghamdi, and Y. M. Lu, Optimal spectral initialization for signal recovery with applications to phase retrieval, IEEE Transactions on Signal Processing **67**, 2347 (2019).

[5] O. Katz, P. Heidmann, M. Fink, and S. Gigan, Non-invasive single-shot imaging through scattering layers and around corners via speckle correlations, Nature photonics **8**, 784 (2014).

[6] C. Moretti and S. Gigan, Readout of fluorescence functional signals through highly scattering tissue, arXiv preprint arXiv:1906.02604 (2019).

[7] M. A. Cox and T. F. Cox, Multidimensional scaling, in *Handbook of data visualization* (Springer, 2008) pp. 315–347.
